# Predictors of morbidity and mortality in patients submitted to cytoreductive surgery plus hyperthermic intraperitoneal chemotherapy for ovarian carcinomatosis: a multicenter study

**DOI:** 10.1515/pp-2020-0139

**Published:** 2020-12-04

**Authors:** Antonio Macrì, Fabio Accarpio, Vincenzo Arcoraci, Francesco Casella, Franco De Cian, Pierandrea De Iaco, Elena Orsenigo, Franco Roviello, Giovanni Scambia, Edoardo Saladino, Marica Galati

**Affiliations:** Department of Human Pathology, University of Messina, Via Consolare Valeria, 98125, Messina, Italy; Cytoreductive Surgery and HIPEC Unit – Department of Surgery “Pietro Valdoni”, University “Sapienza” of Rome, Rome, Italy; Department of Clinical and Experimental Medicine, University of Messina, Messina, Italy; 1st Department of General Surgery, University of Verona, Verona, Italy; Department of Surgery, University of Genoa, Genoa, Italy; Department of Obstetrics and Gynecology, St. Orsola Hospital, University of Bologna, Bologna, Italy; Department of Surgery, San Raffaele Scientific Institute, Milan, Italy; Department of Medicine, Surgery and Neuroscience, University of Siena, Siena, Italy; Department of Obstetrics and Gynecology, Catholic University of the Sacred Heart, Rome, Italy; Papardo Hospital, Messina, Italy; Emergency Surgery Unit, University Hospital, Messina, Italy

**Keywords:** hyperthermic intraperitoneal chemotherapy (HIPEC), morbidity, mortality, ovarian carcinomatosis, predictor

## Abstract

**Objectives:**

The aim of this retrospective study is to assess the incidence of morbidity and mortality related to cytoreductive surgery (CRS) plus hyperthermic intraperitoneal chemotherapy (HIPEC) and to evaluate their predictors, in patients with peritoneal metastasis of ovarian origin.

**Methods:**

A retrospective multicenter study was carried out investigating results from eight Italian institutions. A total of 276 patients met inclusion criteria. Predictors of morbidity and mortality were evaluated with univariate and multivariate analysis.

**Results:**

Overall morbidity was 71.4%, and severe complications occurred in 23.9% of the sample; 60-day mortality was 4.3%. According to univariate logistic regression models, grade 3–4 morbidity was related to Peritoneal Cancer Index (PCI) (OR 1.06; 95% CI 1.02–1.09; p<0.001), number of intraoperative blood transfusions (OR 1.21; 95% CI 1.10–1.34; p<0.001), Completeness of Cytoreduction (CC) score (OR 1.68; 95% CI 1.16–2.44; p=0.006) and number of anastomoses (OR 1.32; 95% CI 1.00–1.73; p=0.046). However, at the multivariate logistic regression analysis, only the number of intraoperative blood transfusions (OR 1.17; 95% CI 1.5–1.30; p=0.004) and PCI (OR 1.04; 95% CI 1.01–1.08; p=0.010) resulted as key predictors of severe morbidity. Furthermore, using multivariate logistic regression model, ECOG score (OR 2.45; 95% CI 1.21–4.93; p=0.012) and the number of severe complications (OR 2.16; 95% CI 1.03–4.52; p=0.042) were recorded as predictors of exitus within 60 days.

**Conclusions:**

The combination of CRS and HIPEC for treating peritoneal metastasis of ovarian origin has acceptable morbidity and mortality and, therefore, it can be considered as an option in selected patients.

## Introduction

Epithelial ovarian cancer (EOC) represents the most frequent cause of death among women suffering from gynecologic malignancies and the fifth cause of cancer-related death in women [1]. Unfortunately, in up to 75% of patients, the disease is diagnosed at an advanced stage, when the presence of peritoneal involvement and distant metastasis has a strong impact on prognosis [2]. Primary surgical cytoreduction (CRS) and cisplatin/taxol-based systemic chemotherapy represent the current standard of care for advanced EOC. Surgery could be defined as the cornerstone procedure, and the absence of residual disease is the strongest independent predictor of survival [2], [3], [4]. The peritoneum is usually the primary site of dissemination in EOC, although, even in case of recurrence, the disease usually remains confined inside the peritoneal cavity for most of its natural history and therefore it can be amenable to local–regional treatment [4].

Notwithstanding systemic carboplatin/taxol-based first-line chemotherapy allows a good response rate, with a high proportion of complete responses, recurrence occurs in up to 70% of stage-III patients [2], [3], [5], [6], [7], [8].

Long-term survival has been reported around 20–30%, and it has been related to disease-free interval [2], [3], [5], [6], [7], [8], [9], [10]. Second-line treatments resulted even less effective, and most patients ultimately die of chemorefractory disease [11].

Platinum sensitivity, in fact, is a very important prognostic factor. Helm et al. analyzed the difference between platinum-resistant and platinum-sensitive patients in terms of response rates and median survival, and have reported, respectively, values of 28 vs. 77% and 6–12 vs. 12–40 months [12].

Management of relapse depends on its platinum-sensitivity [13] and is based on multiple novel targeted-therapies, while the role of secondary cytoreductive surgery is still controversial [14].

Neoadjuvant chemotherapy (NACT) followed by attempting cytoreductive surgery, as first-line treatment for advanced EOC, is a different approach especially for patients not suitable for standard care. Although NACT failed to improve survival, it helped to enhance the rate of optimal cytoreduction [15, 16].

Both controversial results and the prevalent locoregional diffusion of EOC, suggest that intraperitoneal chemotherapy (IPC) could be considered as an useful option in the multimodal management of this disease.

IPC exploits the peritoneal-plasma barrier, and its main advantage is the high concentration of the drug exactly at the intra-abdominal tumor site, as well as the minimal systemic drug exposure with a lower potential toxicity compared to intravenous administration [17]. Despite the benefits of IPC, clinicians are reluctant to adopt this therapy, mostly for its potential greater toxicity, in addiction to catheter-related complications [18].

In view of the foregoing, several institutions worldwide proposed to combine maximal CRS with Hyperthermic Intraperitoneal Chemotherapy (HIPEC) for patients suffering from advanced EOC. Hyperthermia advantages includes a direct tumoricidal effect and the raise of neoplastic cells sensitivity to chemotherapy [19]. The degree of drug penetration into tumor tissues ranges from a few layers of cells to 1–3 mm in depth, therefore, complete CRS is paramount. Despite the promising role of HIPEC + CRS, this approach is associated with risk of surgical complications and systemic toxicity [20].

As previously reported [21], morbidity and mortality are the most comprehensive parameters used to evaluate short-term outcomes of specific procedures and surgical complications are frequently considered as the main reason to modify patient’s management.

## Materials and methods

A total of 397 patients were registered in the database; however, we were able to enroll only 276 due to the incompleteness of the data that the various centers, in a uniform manner, recorded (Table 1).

**Table 1:** Baseline characteristics of 276 patients.

Characteristics	Value, n %
Age, years, median (IQ range)	58 (49.25–65.0)
BMI (kg/m^2^) median (IQ range)	24.2 (22.0–28.0)
ECOG score, n, %	
0	136 (49.3)
1	81 (29.3)
2	47 (17.0)
3	12 (4.3)
ASA score, n, %	
1	49 (17.8)
2	100 (36.2)
3	95 (34.4)
4	32 (11.6)
NACT, n, %	52 (18.8)
Ascites, n, %	80 (29.0)
PCI, median (IQ range)	9.0 (5.0–18.0)

BMI, body mass index; ECOG, Eastern Cooperative Oncology Group; ASA, American Society of Anesthesiologist; NACT, neoadjuvant chemotherapy; PCI, Peritoneal Cancer Index.

A dedicated case report form, created for the study and recorded anonymously in a central database, was used to collect data. Information outside the established rules of quality and completeness were investigated and back-submitted to each participating center in order to receive immediate feedback. Postoperative morbidity was evaluated in accordance with the National Cancer Institute Common Terminology Criteria for Adverse Events (CTCAE) v4.0 [22]. The intraoperative staging of PM was performed following Peritoneal Cancer Index (PCI) and the evaluation of residual disease according to Completeness of Cytoreduction score (CC-score) [23]. The mean PCI was 11.8, with no statistically significant differences between centers. Demographic, clinical and surgical characteristics of each patient were analyzed according to total postoperative major morbidity and mortality.

Patients characteristics as age, body-mass index (BMI), American Society of Anaesthesiologist (ASA) score, Eastern Cooperative Group (ECOG) score and the presence of ascites, as well as NACT, HIPEC technique, chemotherapy treatment scheduled for HIPEC, PCI, number of anastomoses, timing of anastomoses, number of intraoperative blood transfusions, operative time, CC-score, repeated-HIPEC (RE-HIPEC) and up-front approach were evaluated, in order to understand the possible influence of these factors on the occurrence of severe (grade 3–4) complications and postoperative mortality. The incidence of morbidity was calculated as the ratio between the number of patients in which at least one complication occurred and the number of individuals underwent surgical procedures. Mortality was evaluated as patients’ death within 60 days from surgical procedures. All patients underwent a follow-up visit 60 days after surgery.

Due to an abnormal distribution of some numerical variables and low sample size, which did not guarantee valid asymptotic results, a nonparametric approach was used. All results were expressed as median with an interquartile range for continue variables, absolute and percentage frequencies for categorical variables.

Different univariate logistic regression models, using absence of complications as comparative, were used to assess the possible influence of each covariate on morbidity. Moreover, univariate logistic regression models, using absence of death as comparative, were used in order to identify mortality predictors. Every factor resulted significant at the univariate models was also included in multivariate logistic regression models (adjusted OR). Odds ratios (ORs) with 95% confidence interval were calculated for each covariate of interest. Statistical analyses were performed using SPSS.20.0 (IBM Corp. SPSS Statistics).

Peritonectomy was performed as Sugarbaker originally showed in his technique description and therefore through a sequence of maneuvers well codified [24, 25] carried out depending on the extent of the disease [2], [3], [5], [18], [20].

## Results

Demographic, patients’ characteristics and surgical details of the sample are summarized in Tables 1 and 2. The list and incidence of complications are reported in Table 3.

**Table 2:** Surgical details.

Technique of HIPEC	n %
Closed abdomen	213 (77.2)
Coliseum technique	63 (22.8)
Intraoperative transfusion median (IQ range)	3.0 (1.0–4.0)
Treatment n, %	
Cisplatinum + taxol	40 (13.7)
Cisplatinum	136 (49.3)
Oxaliplatinum	40 (14.5)
Cisplatinum + mitomycin	30 (10.9)
Cisplatinum + doxorubicin	26 (9.4)
Other	4 (1.4)
Operative time (min) median (IQ range)	500 (400–607.5)
CC score, n, %	
0	209 (75.7)
1	45 (16.3)
2	17 (6.2)
3	5 (1.8)
Anastomosis, n, %	154 (55.8)
Before HIPEC	120 (43.5)
After HIPEC	34 (12.3)
Number of anastomosis median (IQ range)	1.0 (0.0–2.0)
0	122 (44.2)
1	84 (30.4)
2	50 (18.1)
3	17 (6.2)
4	3 (1.1)
ICU, day, mean (range)	2.1 (0.0–18.0)
Hospital stay (day) median (IQ range)	14.0 (10.0–20.0)
Re-HIPEC	25 (9.1)
UP front	98 (35.5)
Mortality n, %	12 (4.3)
Compl.1–4 n, %	197 (71.4)
Compl.1–2 n, %	133 (47.5)
Compl.3–4 n, %	66 (23.9)

HIPEC, hyperthermic intraperitoneal chemotherapy; CC score, Completeness of Cytoreduction score; ICU, intensive care unit.

**Table 3:** List of complications.

	n	%
**Blood disorders**		
Anemia	**21**	**7.61**
Leukopenia	**2**	**0.72**
Thrombocytopenia	**4**	**1.45**
**Cardiac disorders**		
Arrhythmia	–	
Myocardial infarction	**2**	**0.72**
**Gastrointestinal disorders**		
Intestinal perforation	**8**	**2.90**
Hemoperitoneum	**8**	**2.90**
Anastomotic leakage	**1**	**0.36**
Pancreatic fistula	–	
GI haemorrhage	**4**	**1.45**
Abdominal abscess	**5**	**1.81**
Stoma complications	**2**	**0.72**
**General disorders**		
Nausea	–	
Emesis	–	
Diarrhea	–	
Postoperative ileus	**1**	**0.36**
Gastric stasis	–	
Hyperpyrexia	–	
**Hepatobiliarypancreatic disorders**		
Pancreatitis	**8**	**2.90**
Biliary complications	–	
**Infections**		
Sepsis	**16**	**5.80**
MOF	**6**	**2.17**
**Nervous system disorders**		
Neurological complications	–	
**Respiratory, thoracic and mediastinal disorder**		
Pleural effusion	**6**	**2.17**
Pulmonary oedema	–	
Pulmonary embolism	**6**	**2.17**
Acute respiratory distress syndrome	**4**	**1.45**
Pneumonia	–	
Pneumothorax	**2**	**0.72**
Diaphragmatic perforation	–	
**Renal and urinary disorders**		
Urinary infection	–	
Renal failure	**2**	**0.72**
Urinary leakage	–	
**Skin and subcutaneous disorders**		
Wound infection	**2**	**0.72**
**Vascular disorders**		
DVP	–	
DIC	–	

GI, gastrointestinal; MOF, multiple organ failure; DVP, deep venous thrombosis; DIC, disseminated intravascular coagulation.

Primary and recurrent ovarian cancer patients were 86 and 190, respectively. In the first group, 61 patients (70.9%) were submitted to upfront HIPEC, while 25 (29.1%) to interval surgery plus HIPEC. In the second one, 128 (67.4%) were submitted to upfront HIPEC, 37 (19.5%) to interval surgery plus HIPEC and 25 (13.1%) to salvage HIPEC.

No differences resulted in the median age and BMI of patients with or without severe morbidity. Comparable were also the frequency of ascites, ECOG score, and ASA score in both groups.

PCI was significantly higher in patients that experienced severe morbidity [16.5 (IQ 5.7–24.0) vs. 9.0 (IQ 4.0–16.0)], without statistically significant variations between the centers.

Severe complications affected 15.9 and 26.3% of patients underwent to Coliseum and Closed abdomen HIPEC techniques, respectively. In patients with severe complications (SC group), the median number of anastomosis and intraoperative blood transfusions were 1.0(IQ 0.0–2.0) and 3.0 (IQ 2.0–6.0), respectively, vs. 1.0 (IQ 0.0–1.0) and 2.0 (IQ 0.0–4.0) recorded in patients with low morbidity (LM group).

Operative time was 520.0 min (IQ 420.0–660.0) in the SC group and 500.0 min (IQ 400.0–600.0) in the LM group. CC score was 0 in 59.1% (95% CI 47.2–71.0%) and 1 in 30.3% (95% CI 19.2–41.4%) in SC group, while it was 0 in 81% (95% CI 75.6–86.3%) and 1 in 11.9% (95% CI 7.5–16.3%) LM group. Concerning HIPEC chemotherapy, severe complications affected 27.9% (95% CI 20.4–35.5%) of patients treated with CDDP, 27.5% (95% CI 13.7–41.3%) with OX, 23.1% (95% CI 6.9–39.3%) with CDDP plus DOX, 16.7% (95% CI 3.3–30.0%) with CDDP plus MMC and 12.5% (95% CI 2.3–22.7%) with CDDP plus Taxol.

Within 25 patients who already had previous HIPEC (RE-HIPEC), 4 showed severe complications (16.0%; 95% CI 1.6–30.4%). In patients submitted to up-front CRS + HIPEC, 18 out of 98 patients (18.4%; 95% CI 10.7–26.0%) were in SC group.

According to univariate logistic regression models, severe morbidity depends on PCI (OR 1.06; 95% CI 1.02–1.09; p<0.001), number of intraoperative blood transfusions (OR 1.21; 95% CI 1.10–1.34; p<0.001), intestinal anastomoses (OR 1.32; 95% CI 1.00–1.73; p=0.046) and CC score (OR 1.68; 95% CI 1.16–2.44; p=0.006). However, in the multivariate logistic regression analysis, only the number of intraoperative blood transfusions (OR 1.17; 95% CI 1.5–1.30; p=0.004) and PCI (OR 1.04; 95% CI 1.01–1.08; p=0.010) resulted as the key predictors of severe morbidity.

Predictive factors of severe complications are described in Table 4.

**Table 4:** Predictors of severe morbidity assessed by univariate and multivariate logistic regression analysis.

Independent variables	OR_crude_ (CI 95%)	p-Value	OR_adjusted_ (CI 95%)	p-Value
**Age**	1.01 (0.98–1.03)	0.629	--	--
**BMI**	1.00 (0.92–1.05)	0.633	--	--
**ASA**	0.80 (0.59–1.08)	0.150	--	--
**ECOG**	1.20 (0.89–1.63)	0.230	--	--
**Ascites**	0.99 (0.54–1.82)	0.968	--	--
**PCI**	1.06 (1.02–1.09)	<0.001	1.04 (1.01–1.08)	0.010
**NACT**	0.94 (0.46–1.93)	0.875	--	--
**HIPEC**				
Closed abdomen	1			
Coliseum technique	0.53 (0.25–1.11)	0.092	--	--
Semiclosed abdomen	--	--	--	--
**Anastomosis**	1.67 (0.94–2.96)	0.081	--	--
**Number of anastomosis**	1.32 (1.00–1.73)	0.046	1.18 (0.88–1.57)	0.268
**Timing of anastomosis**	0.37 (0.13–1.04)	0.058	--	--
**Number of transfusions**	1.21 (1.10–1.34)	<0.001	1.17 (1.05–1.30 )	0.004
**Operative time**	1.00 (0.99–1.00)	0.509	--	--
**CC score**	1.68 (1.16–2.44)	0.006	1.24 (0.77–2.00)	0.366
**Treatment**				
Cisplatinum + taxol	1			
Cisplatinum	2.71 (0.98–7.45)	0.052	--	--
Oxaliplatinum	2.65 (0.83–8.52)	0.101	--	--
Cisplatinum + mitomycin	1.40 (0.37–5.35)	0.623	--	--
Cisplatinum + doxorubicin	2.10 (0.57–7.77)	0.266	--	--
**RE-HIPEC**	0.58 (0.19–1.75)	0.336	--	--
**Up front**	0.61 (0.33–1.12)	0.111	--	--

BMI, body mass index; ASA, American Society of Anesthesiologists; ECOG, Eastern Cooperative Oncology Group; PCI, Peritoneal Cancer Index; NACT, neoadjuvant chemotherapy; HIPEC, hyperthermic intraperitoneal chemotherapy; CC score, Completeness of Cytoreduction score.

Concerning the conditions affecting mortality, no differences resulted in the median age of patients dead or alive, as well as BMI, frequency of ascites and ASA score. ECOG score and PCI were significantly higher in Dead Patients group (DP) compared to Alive Patients group (AP). Coliseum and Closed abdomen HIPEC techniques were related to DP in 4.8% (95% CI –0.5 to 10.0%) and 4.2% (95% CI 1.5–6.9%) in AP, respectively. The median number of anastomosis, intraoperative blood transfusions and CC score were similar in both groups. Operative time was 600.0 min (IQ range 435.0–702.5) in the DP group and 500.0 min (400.0–600.0) in AP one.

Regarding anticancer drugs used during HIPEC, death, respectively, occurred in 7.7% (95% CI −2.6 to 17.9%), 5.1% (95% CI 1.4–8.9%) and 5.0% (95% CI −1.8 to 11.8%) of patients treated with CDDP + DOX, CDDP and CDDP + TAX. No patient died among those treated with OX and CDDP + MMC. Within 25 patients who underwent RE-HIPEC, only 1 patient died (4.0%; 95% CI −3.7–11.7%); 5 out of 98 patients underwent to up-front treatment died (5.1%; 95% CI 0.7–9.5%).

According to univariate logistic regression, mortality was related to ECOG score (OR 2.51; 95% CI 1.38–4.55; p=0.002), PCI (OR 1.10; 95% CI 1.04–1.17; p=0.001), number of intraoperative transfusions (OR 1.35; 95% CI 1.13–1.59; p=0.001), CC-score (OR 2.30, 95% CI 1.27–4.14; p=0.006) and number of severe complications (OR 3.41; 95% 2.06–5.64; p=0.001).

Nevertheless, after inclusion in the multivariate logistic regression, only ECOG score (OR 2.45; 95% CI 1.21–4.93; p=0.012) and number of severe complications (OR 2.16; 95% CI 1.03–4.52; p=0.042) resulted as the real predictors of exitus within 60 days. Predictive factors of mortality are described in Table 5.

**Table 5:** Predictors of mortality assessed by univariate and multivariate logistic regression analysis.

Independent variables	OR_crude_ (CI 95%)	p-Value	OR_adjusted_ (CI 95%)	p-Value
**Age**	1.02 (0.97–1.08)	0.369	--	--
**BMI**	1.04 (0.92–1.18)	0.549	--	--
**ASA**	1.14 (0.60–2.15)	0.693	--	--
**ECOG**	2.51 (1.38–4.55)	0.002	2.45 (1.21–4.93)	0.012
**Ascites**	1.24 (0.36–4.23)	0.735	--	--
**PCI**	1.10 (1.04–1.17)	0.001	1.02 (0.92–1.12)	0.713
**NACT**	1.46 (0.38–5.60)	0.579	--	--
**HIPEC**				
Closed abdomen	1			
Coliseum technique	1.13 (0.30–4.32)	0.855	--	--
Semiclosed abdomen	--	--	--	--
**Anastomosis**	2.46 (0.65–9.30)	0.184	--	--
**Number of anastomosis**	1.52 (0.90–2.56)	0.119	--	--
**Number of transfusions**	1.34 (1.13–1.59)	0.001	1.21 (0.97–1.50)	0.083
**Operative time**	1.00 (1.00–1.01)	0.576	--	--
**CC score**	2.30 (1.27–4.14)	0.006	1.76 (0.73–4.24)	0.209
**Treatment**				
Cisplatinum + taxol	1			
Cisplatinum	1.03 (0.21–5.17)	0.970	--	--
Oxaliplatinum	--	--	--	--
Cisplatinum + mitomycin	--	--	--	--
Cisplatinum + doxorubicin	1.58 (0.21–12.00)	0.657	--	--
**RE-HIPEC**	0.91 (0.11–7.35)	0.929	--	--
**Up front**	1.31 (0.41–4.25)	0.649	--	--
**N. Compl 3–4**	3.41 (2.06–5.64)	<0.001	2.16 (1.03–4.52)	0.042

BMI, body mass index; ASA, American Society of Anesthesiologists; ECOG, Eastern Cooperative Oncology Group; PCI, Peritoneal Cancer Index; NACT, neoadjuvant chemotherapy; HIPEC, hyperthermic intraperitoneal chemotherapy; CC score, Completeness of Cytoreduction score.

## Discussion

More than 75% of patients suffering from EOC is diagnosed at an advanced stage, with an overall 5-year survival less than 20% [26]. Currently, optimal CRS with intravenous perioperative chemotherapy, with a combination of platinum- and taxane-based drugs, is the main frontline treatment [5, 27]. When primary CRS is not feasible, 3 cycles of NACT followed by interval CRS plus 3 cycles of adjuvant chemotherapy could be the best option. In a randomized trial comparing primary CRS followed by chemotherapy vs. NACT with interval CRS, no difference in survival was found, although the second group experienced a better QoL during the first year [27].

HIPEC is an alternative method for administering intraperitoneal chemotherapy immediately after peritonectomy. It takes advantage from the synergism between hyperthermia and antiblastic drugs, furthermore, it provides a better distribution on peritoneal surface, as well as better penetration in the invisible microscopic residual tumor. Moreover, HIPEC can exceed the common limits of the post-operative intraperitoneal chemotherapy, as incomplete coverage of the peritoneum, suboptimal treatment, catheter-related complications, frequent impossibility to complete 6 cycles of chemotherapy, need for catheter removal [28, 29], worse QoL during the first year [30].

The association of CRS + HIPEC is particularly useful in EOC because of its locoregional diffusion without initial distant metastases, frequent chemosensitivity and adequate response to postoperative intraperitoneal chemotherapy [27].

Spiliotis et al. published the first phase III randomized prospective study, involving 120 patients with stage IIIc or IV recurrent EOC, who underwent CRS + HIPEC vs. CRS both along with adjuvant chemotherapy. They reported a significant increase in mean survival in the former group (26.7 vs. 13.4 months, p<0.006), with 3-year survival of 75 vs. 18% (p<0.01). Moreover, the differences in survival rates between patients with and without platinum resistance, was not significant in the HIPEC group while it was significant in the systemic chemotherapy group [31].

Although CRS + HIPEC may improve survival in patients with ovarian carcinomatosis, this option is not commonly adopted, mainly because of its relevant morbidity and mortality, also if Van Driel et al. in a very important study [32], demonstrated that, in patients with stage III epithelial ovarian cancer, the addition of HIPEC to interval cytoreductive surgery resulted in longer recurrence-free survival and overall survival than surgery alone and did not result in higher rates of side effects.

Major morbidity has been previously reported ranging from 12 to 57% in high-volume centers [21]. Improvements are usually related to the mandatory learning curve needed to minimize mortality and morbidity [33]. Therefore, the evaluation of morbidity, mortality and the identification of risk factors for postoperative complications as well as oncological outcomes represent a major concern.

Despite CRS + HIPEC allow to obtain good results, the whole procedure is complex and time-consuming, and any survival benefit must be evaluated compared to morbidity and mortality.

Recently, Gonzalez Bayon treated 42 patients suffering from EOC with CRS + HIPEC either at first diagnosis or at first recurrence, and at second or subsequent recurrence. He found a severe morbidity rate of 26%, postoperative mortality was nearly 7%, respectively. At multivariate analysis, PCI, CC-score and the use of cisplatin were factors related to morbidity [34].

In a French study on 566 patients affected by advanced or recurrent EOC, CC-0 rate was around 75%, overall survival was 35.4 and 45.7 months for advanced and recurrent EOC, while severe morbidity and mortality were 31.3 and 0.8%, respectively [35].

In a multicenter prospective study of 54 patients treated with HIPEC at various time points during their management, Coccolini and colleagues reported a severe morbidity rate of 35.2% and a mortality of 5.6%. At the univariate and multivariate analysis, they found more severe postoperative complications in patients who underwent HIPEC as an upfront treatment [36].

In 2016, Muñoz-Casares et al. analyzed 218 patients treated with CRS + HIPEC for peritoneal metastasis from primary or recurrent EOC. Overall morbidity was 35%, with a severe morbidity of 15%, while mortality was 1.4% [37].

Desantis et al. [38] reported 400 CRS + HIPEC on 356 patients, of which 50% was affected by ovarian carcinomatosis. Based on CTCAE score they documented a grade III-IV morbidity of 12.5% and a mortality of 1%. At the univariate analysis, several factors seemed to be predictors of severe morbidity, as well as WHO performance status (p=0.04), perioperative blood transfusion (p=0.006), number of gastrointestinal anastomoses (p<0.0001) and the CC-score 0 (p=0.0013). Nonetheless, at multivariate analysis, only the number of anastomoses (>1) were significantly associated with morbidity and mortality (OR 2.8; 95% CI 1.09–7.2, p=0.032) [38].

In a recent Italian multicenter trial involving 511 patients with advanced EOC, treated with CRS + HIPEC, major morbidity was nearly 45% and mortality was 2.5%. Multivariate logistic regression analysis identified a CC-score>0 and the need for more than 4 blood transfusions during surgery as significant risk factors for major complications [39]. In our series, overall morbidity was 71.4%, with a rate of severe complications of 23.9%, while mortality within 60 days was 4.3%.

After inclusion in univariate and multivariate logistic regression models, we observed that age, BMI, ECOG, ASA, NACT, ascites, type of HIPEC (closed vs. coliseum), Upfront HIPEC, RE-HIPEC, chemotherapeutic treatment, operative time, ICU and hospital stay do not influence severe morbidity.

Despite the univariate logistic regression identified PCI, number of intraoperative blood transfusion, CC-score and number of anastomoses as predictors of severe morbidity, after their inclusion in the multivariate logistic regression model only PCI and the number of intraoperative blood transfusions resulted as the key predictors of severe morbidity.

Our results confirm that higher the PCI, greater is the risk of severe complications, indeed, our patients had 4% risk to develop severe complications for each 1-unit increase of the PCI score. Probably it reflects the degree of diffusion, the aggressiveness of the disease and the complexity of the surgery required to achieve CRS.

The median number of intraoperative blood transfusions was 3.0 (IQ range 2.0–6.0) and it was higher (OR 1.21; 95% CI 1.10–1.34; p<0.001) in patients with 3–4 morbidity, reflecting therefore the complexity of the cytoreductive surgery.

CC score was 0 in 59.1% and 1 in 30.3% of patients with severe complications, while CC score was 0 in 81.0% and 1 in 11.9% of patients without severe complications. A higher CC score value (OR 1.68; 95% CI 1.16–2.44; p=0.006) was correlated with an higher risk of 3–4 complications. Thus, an elevated CC-score identifies the inability to achieve an optimal CRS due to the extension of the PM, and consequently the high aggressivity and diffusion of the PM.

The median number of anastomoses was 1.0 (IQ range 0.0–2.0). In 154/276 patients (55.8%) were performed at least one anastomosis and 66/154 (42.8%) developed severe complications. The frequency of severe complications was higher among the patients in which anastomoses were performed (42.8% vs. 18.9%), moreover the frequency of severe complications increases with the number of anastomoses and this correlation resulted statistically significant (OR 1.32; 95% CI 1.00–1.73; p=0.046) in univariate analysis.

Even more, our data underline that the risk to develop total complications is lower when the anastomoses are performed after HIPEC but this difference was statistically significant only in univariate analysis (OR 0:39; 95% CI 0.18–0.88; p=0.025). This item is however interesting and needs further investigations.

The anastomoses performed after HIPEC can be less exposed in fewer cases to leakage or dehiscence, probably for the less exposure to the stress forces generated during the perfusion and therefore in the anastomoses performed before HIPEC the mechanical traction of viscera during the perfusion could impair their integrity.

Furthermore, in the anastomoses performed after HIPEC, the potential adverse effects of heat and chemotherapy on the suture healing probably could be lower. The influence of CHT on the suture healing depends on the type of drug. In animals’ studies, anastomotic healing can be impaired by intraperitoneal MMC but not by 5-FU or paclitaxel. Local hyperthermia in itself has no adverse effect on rat anastomotic healing [39].

At the light of the data of the literature and of our results, to obtain a smaller morbidity is therefore necessary an appropriate selection of patients. The extension of the PM represents one of the major prognosis factors, because it can require a greater extent of the surgery.

In our experience, according to univariate logistic regression, mortality was related to ECOG score, PCI, number of intraoperative transfusions, CC-score and number of severe complications.

Nevertheless, after inclusion in the multivariate logistic regression, only ECOG score and number of severe complications resulted real predictors of exitus within 60 days.

At the light of these results, to reduce mortality, a careful patients’ selection appears essential.

## Conclusions

The analysis of the results of our experience, compared to those of the largest published experiences (Table 6), shows morbidity and mortality within the range. The predictors of morbidity emerging from our case histories, number of intraoperative transfusions and PCI, are also shared by other authors [34, 40].

**Table 6:** Comparison between our experience and literature data.

Author	PTS, n	1–2 morbidity	3–4 morbidity	Mortality	3–4 morbidity predictors (multivariate analyses)	Morbidity predictors (multivariate analyses)
Gonzales Bayon 2013	42		26%	7%		
Bakrin 2013	566		31.3	0.8	EOC treated as first line (OR 1.7; p 0.008) PCI more than 8 (OR 2.17; p 0.003) CC-1 and CC-2 (OR 2.06; p 0.031) use of cisplatin (OR 3.08; p 0.002)	
Coccolini 2014	54	60.2	35.2	5.6	EOC treated as first line	
Munoz Casares 2016	218	21.1	13.8	1.4		
Desantis 2015	197		12.5	1	Number of anastomoses >1 (OR 2.8; 95% CI 1.09–7.2, p=0.032.)	
Franko 2008	65				Number of anastomoses (p=0.032)	
					Intraoperative blood loss (p=0.034)	
Di Giorgio 2017	511	26.8	17.4	2.5	CC score >0 (p=0.013, OR 0.24982, 95%CI 0.29197–2.48208)	
					Intraoperative blood transfusions >4 (p=0.002, OR 0.35323, 95%CI 0.37162–1.70963)	
This experience	276	47.5	23.9	4.3	Number of intraoperative blood transfusions (OR 1.17; 95% CI 1.5–1.30; p=0.004)	ECOG score (OR 2.45; 95% CI 1.21–4.93; p=0.012)
					PCI (OR 1.04; 95% CI 1.01–1.08; p=0.010)	Number of severe complications (OR 2.16; 95% CI 1.03–4.52; p=0.042).

PTS, patients; EOC, epithelial ovarian cancer; CC, Completeness of Cytoreduction score; ECOG, Eastern Cooperative Oncology Group; PCI, Peritoneal Cancer Index.

In particular, the performance status and the number of severe complications were predictors of mortality. At the light of all the above considerations, the incidence of complications appears to be related mainly to factors associated with the extension of the disease and, therefore, to the extent of surgery, while mortality appears to be mainly dependent on the clinical condition of the patient.

Our study, confirming the feasibility of CRS plus HIPEC, highlights the importance of a correct and strict patient selection.

The ideal patient must therefore have a good PS and be affected by an ovarian carcinomatosis with low PCI. Patients with high performance status should not be candidate to this type of surgery. A strict follow up of patients with advanced EOC is mandatory to identify, as early as possible, a peritoneal recurrence. Furthermore, trained surgeons and multidisciplinary teams are needed to minimize blood loss and anastomotic leaks and to achieve complete tumor excision.
